# Diet, obesity and colorectal carcinoma risk: results from a national cancer registry-based middle-eastern study

**DOI:** 10.1186/s12885-018-5132-9

**Published:** 2018-12-07

**Authors:** Nourah Alsheridah, Saeed Akhtar

**Affiliations:** Department of Community Medicine and Behavioural Sciences, Faculty of Medicine, Kuwait University, P.O. Box 24923, Safat, 13110 Kuwait

**Keywords:** Colorectal cancer, Matched case-control study, Conditional logistic regression, Risk factors

## Abstract

**Background:**

Cancer of colon and rectum (colorectal) is one of the most common cancers worldwide. There is a scarcity of published data on the risk factors for colorectal cancer (CRC) from the Middle-Eastern countries specifically in Kuwait. Therefore, this matched case-control study sought to examine the risk factors associated with CRC in Kuwait.

**Methods:**

One hundred and three histopathologically confirmed colorectal cancer cases were recruited from Kuwait Cancer Control Centre Registry. Two hundred and six controls matched with cases (2:1 ratio) on age, gender and nationality were selected from medical, ophthalmology, orthopedic and/ or surgical out-patient clinics at three main general hospitals in Kuwait. A structured questionnaire was used to collect the data from cases and controls through face-to-face interview. Adjusted matched odds ratios (mOR_adj_) and their 95% confidence intervals (CI) were estimated using a multivariable conditional logistic regression model.

**Results:**

Multivariable conditional logistic regression model showed that cases were 4.3 times more likely to have had attainted obesity (BMI ≥ 30) in their lifetime compared to controls (mOR_adj_ = 4.3; 95% CI: 1.6–11.4). Compared to controls, cases rarely consumed fruits and vegetable (mOR_adj_ = 20.8; 95% CI: 4.4–99.5), tended to consume red meat 2–3 times a week (mOR_adj_ = 3.8; 95% CI: 1.6–8.7) or more than 4 times a week (mOR_adj_ = 9.4; 95% CI: 2.5–35.4). Reportedly cases compared to controls frequently (nearly every week) suffered from constipation (mOR_adj_ = 5.6; 95% CI: 1.9–16.5). However, CRC cases were less likely than controls to have been diagnosed in the past with hypercholesterolemia (mOR_adj_ = 0.3; 95% CI: 0.2–0.7) or diabetes mellitus type II (mOR_adj_ = 0.4; 95% CI: 0.2–0.8).

**Conclusions:**

Obesity, excessive red meat consumption and infrequent fruits/vegetables intake were associated with an increased CRC risk**.** Overcoming identified pitfalls in dietary pattern and maintenance of healthy weight may help minimize CRC risk in Kuwait and perhaps other countries in the region. Further studies on genetic basis in conjunction with life styles and dietary factors may unravel their joint contributions to CRC risk and furnish tools for curtailing CRC risk in this and other similar populations.

## Introduction

Colorectal cancer (CRC) is the third most commonly diagnosed malignancy and the fourth leading cause of cancer deaths in the world. About 1.4 million new cases are diagnosed every year and this figure is likely to rise to 2.4 million by 2035 [1–3]. Additionally, more than half a million people die of CRC every year, which constitutes approximately 8% of all cancer-related deaths worldwide [4–6]. In developed countries, these malignancies rank second both in incidence and mortality, compared with fifth in relatively less developed countries [7, 8]. Geographically, CRC incidence rates vary across the world’s regions from high in North America, Australia, UK and parts of Europe to low in Asian countries [5]. China and Japan are considered to have the highest incidence rates of CRC in Asia [9]. These worldwide variations in the CRC incidence rates are mostly attributed to inappropriate lifestyle and behavioral patterns [7]. Epidemiological studies have suggested that, aging, family history of CRC, smoking, alcohol drinking, high caloric intake, physical inactivity, sedentary lifestyle, obesity, and diabetes are some of the risk factors for CRC [7, 10]. Western lifestyle often leads to a host of morbid conditions (e.g. diabetes, hypertension, dyslipidemia, coronary heart disease, gallbladder disease, arthritis, and constipation), which tend to aggregate in CRC patients [11]. Additionally, educational attainment, socioeconomic conditions and menopausal status in women reportedly contribute to spatial variation in CRC risk [5, 12].

There is a paucity of published data on CRC risk and associated factors from the Middle-Eastern countries. In Kuwait, CRC is the second leading cause of cancer related morbidity and mortality both among males and females [13]. Furthermore, the CRC incident rate (per 100,000 individuals) in Kuwait has increased from192 to 229 recorded during 2012 and 2015 respectively. This increase in CRC incidence rates (per 100,000 individuals) was more pronounced in females (115 to 131) than males (77 to 98) [13]. Some of the aforementioned risk factors for CRC such as obesity, physical inactivity, diabetes and family history are highly prevalent in Kuwait [14]. Furthermore, for the past few decades Kuwaiti population has been rapidly adapting western lifestyles and dietary patterns characterized by excessive consumption of red meat, processed meat, diet high in saturated fat, reduced consumption of fruits and vegetables, physical inactivity, and overweight/ obesity [5, 14, 15]. However, these factors have been scarcely evaluated for their impact on CRC risk in Middle-Eastern countries including Kuwait. Therefore, this matched case-control study was designed to examine the role of diet, lifestyle attributes, physical inactivity, overweight/ obesity and family history in CRC risk in Kuwait population.

## Methods and participants

### Setting and study design

Administratively Kuwait is divided into six governorates and each governorate has well defined area, its population and comprises several demarcated districts. Medical services in each governorate comprise a network of primary health care clinics and a general public hospital. In addition, there are centralized specialty hospitals, including the Kuwait Cancer Control Centre (KCCC). KCCC is the specialized cancer treatment hospital equipped with modern facilities for cancer diagnosis, treatment and follow-up. Nearly all suspected cancer patients are referred to KCCC. Cancer patients which are initially diagnosed and/or treated elsewhere are also referred to KCCC for further follow-up. Health care at all levels is provided free of charge by the government to the nationals, whereas, migrant residents have to pay nominal fee to avail medical services [16, 17].

In this evaluation, a hospital-based matched case-control study design was implemented to identify the potential risk factors associated with the CRC risk. Data collection was carried out from July through September 30, 2017. CRC cases diagnosed between February 1, 2016 and July 31, 2017 and registered with KCCC were enrolled. The controls were selected from the general medical, surgical, orthopedic, and ophthalmology out-patient clinics of three main general hospitals (i.e. Amiri hospital, Al Asima; Mubarak Al Kabeer hospital, Hawally; and Farwaniyah hospital, Farwaniyah) of Kuwait.

### Case definition, inclusion and exclusion criteria

Patients with histopathologically confirmed CRC (International Classification of Disease for Oncology (ICD-O) codes: C18.0–18.9, C19, and C20), and treated by gastroenterologist and/or oncologist were enrolled as cases. Patients with previous diagnosis of cancer at any other site of the body, prior history of inflammatory bowel disease, familial adenomatous polyposis and/ or severely ill were excluded.

### Control definition, inclusion and exclusion criteria

Two control subjects were matched to each case by age (± 3 years), gender, and nationality (Kuwaiti, non-Kuwaiti Arab, and non-Kuwaiti non-Arab). Individuals were eligible for enrollment as controls if they did not have a prior history of confirmed CRC and/or cancer of any site of the body and visiting outpatient clinics in one of above noted hospitals with minor complaints (minor trauma/injuries, upper respiratory tract infections, skin rash/infection, headache, etc.). Controls suffering from acute morbidity or expatriates on short visit to Kuwait were excluded.

### Exposures’ assessment

A structured and pretested questionnaire was used to collect the data from cases and controls through face-to-face interview by a trained interviewer. The questionnaire comprised questions which were grouped in two sections i.e. a) sociodemographic characteristics including nationality, governorate of residence, age, gender, marital status, education level, occupation; b) potential exposures including CRC family history, smoking, height, weight, physical activity, (assessed as metabolic equivalents - METs based on responses to 16 questions) [18], dietary pattern (frequency of consumption of dietary items as responses to five questions), histopathological data from medical files at KCCC Registry (for cases). For two controls, a ‘pseudo-diagnosis’ date was determined (i.e. the date on which the control subjects were of the same age as their matching case). Questions on exposures’ assessment were asked both from cases and controls for the period prior to their diagnosis/ pseudo-diagnosis.

### Sample size

This matched case-control study was designed to enroll 100 CRC cases and 200 CRC-free matched controls (1:2 ratio) to relate most of the potential exposures (having a prevalence of at least 0.15 among controls) with outcome variable with an odds ratio (OR) 2.5 or higher assuming 80% study power (1-β), 5% level of significance (α) and 0.2 as a correlation coefficient for exposure(s) between cases and their matched controls.

### Data analysis

Descriptive statistics of sociodemographic variables were computed to characterize the cases and controls. Univariable relationship of each of the socio-demographics and other potential risk factors with CRC status was tested for statistical significance using McNemar’s test and strength of univariable association was quantified by using simple conditional logistic regression analysis. The variables significantly (*p* ≤ 0.15) related with CRC status on univariable analysis were considered for possible inclusion in multivariable conditional logistic regression analysis. The variables independently and significantly (*p* < 0.05) related to the CRC status were retained in the final model. Adjusted matched ORs (mOR_adjsuted_) and their 95% confidence intervals (CIs) were used to interpret the final model.

### Ethics

Before the interview, a written informed consent was sought both from cases and controls after explaining the study objectives. Confidentiality of the collected data was assured to the participants. The study protocol was approved by the institutional ethics review committee and the Research Ethics Committee at the Ministry of Health, Kuwait.

## Results

### Characteristics of the sample

The final study sample included 103 cases and their 206 age (± 3 years), gender and nationality matched controls. More than 90% of cases and controls were ever married. More cases (38.8%) than controls (24.8%) had education less than a high school and in contrast more controls (14.1%) than cases (5.8%) had graduate level of education. Cases and controls did not differ meaningful on the distributions of employment status and monthly income (Kuwaiti Dinar) (Table 1). McNemar’s test revealed the variables significantly (*p* ≤ 0.150) associated with CRC status included family history of CRC, history of diabetes mellitus type II, history of hypercholesterolemia, constipation, consumption of red meat, fruits and vegetables, total physical activity (METs/ week) and WHO recommendations on physical activity (Table 2).

**Table 1:** Characteristics of study participants in a hospital-based matched case-control study of colorectal cancer (CRC) in Kuwait

Characteristic	Cases (*n* = 103) No. (%)	Controls (*n* = 206) No. (%)
Age at study enrollment (year)		
25–40	12 (11.7)	28 (13.6)
41–50	28 (27.2)	52 (25.2)
51–60	30 (29.1)	56 (27.2)
61–70	20 (19.4)	44 (21.4)
71–80	13 (12.6)	26 (12.6)
Gender		
Female	47 (45.6)	94 (45.6)
Male	56 (54.4)	112 (54.4)
Nationality		
Kuwaiti	38 (36.9)	76 (36.9)
Non-Kuwaiti Arab	39 (37.9)	78 (37.9)
Non-Kuwaiti Non-Arab	26 (25.2)	52 (25.2)
Marital status		
Never married	5 (4.9)	14 (6.8)
Ever married	98 (95.1)	192 (93.2)
Education level		
< High-school	40 (38.8)	51 (24.8)
High-school/diploma	27 (26.2)	68 (33.0)
Undergraduate	30 (29.1)	58 (28.2)
Graduate	6 (5.8)	29 (14.1)
Employment Status		
Employed	65 (63.1)	123 (59.7)
Unemployed	38 (36.9)	83 (40.3)
Salary (K.D.)		
< 150	20 (19.4)	30 (14.6)
150–300	14 (13.6)	35 (17)
301–500	11 (10.7)	27 (13.1)
501–1000	28 (27.2)	59 (28.6)
> 1000	30 (29.1)	55 (26.7)
Governorate residency		
Capital	13 (12.6)	60 (29.1)
Hawalley	30 (29.1)	46 (22.3)
Jahra	10 (9.7)	6 (2.9)
Ahmadi	13 (12.6)	4 (1.9)
Mubarak Al-Kabir	7 (6.8)	3 (1.5)
Farwaniyah	30 (29.1)	87 (42.2)

**Table 2:** Chi-squared analysis of association of various risk factors with colorectal cancer status in a hospital-based matched case-control study, Kuwait

Exposure	Cases (n = 103) No. (%)	Controls (n = 206) No. (%)	*p*-value^*^
Smoking Cigarette			0.324
Yes	29 (28.2)	52 (25.2)	
No	74 (71.8)	154 (74.8)	
Drinking alcohol			0.359
Yes	12 (11.7)	18 (8.7)	
No	91 (88.3)	188 (91.3)	
Smoking Hookah			0.503
Yes	13 (12.6)	20 (9.7)	
No	90 (87.4)	186 (90.3)	
Family history with CRC			0.210
Yes	18 (17.5)	22 (10.7)	
No	85 (82.5)	184 (89.3)	
History with T2DM			0.002
Yes	19 (18.4)	82 (39.8)	
No	84 (81.6)	124 (60.2)	
History with hypercholesterolemia			0.001
Yes	23 (22.3)	89 (43.2)	
No	80 (77.7)	117 (56.8)	
Use of NSID			0.401
Yes	35 (34.0)	73 (35.4)	
No	68 (66.0)	133 (64.6)	
Post-menopausal hormone therapy (only females)			0.999
Yes	8 (17.0)	14 (14.9)	
No	39 (83.0)	80 (85.1)	
Constipation			0.156^**^
Never	37 (35.9)	86 (41.7)	
Rarely	16 (15.5)	63 (30.6)	
Once in a month	21 (20.4)	32 (15.5)	
Every week	29 (28.2)	25 (12.1)	
Red meat intake			< 0.001^**^
< 1 day/week	17 (16.5)	92 (44.7)	
1–3 days/week	65 (63.1)	100 (48.5)	
4–6 days/week	21 (20.4)	14 (6.8)	
Milk (glass)			0.831^**^
Rarely (never/< 1 glass)	29 (28.2)	57 (27.7)	
Often	29 (28.2)	62 (30.1)	
Everyday	45 (43.7)	87 (42.2)	
Fruits and vegetables intake (day(s)/week)			< 0.001^**^
Rarely (never/< 1 a day)	16 (15.5)	4 (1.9)	
Often	43 (41.7)	57 (27.7)	
Everyday	44 (42.7)	145 (70.4)	
Cheese intake (day(s)/week)			0.074^**^
< 1 day/week	18 (17.5)	50 (24.3)	
1–6 days/week	35 (34.0)	79 (38.3)	
Everyday	50 (48.5)	77 (37.4)	
Egg(s) intake (day(s)/week)			0.073^**^
< 1 day/week	14 (13.6)	59 (28.6)	
1–6 days/week	75 (72.8)	129 (62.6)	
Everyday	14 (13.6)	18 (8.7)	
Total physical activity per week (METs/week) ^a^			0.033
≤ median (520)	63 (61.2)	95 (46.1)	
> median (520)	40 (38.8)	111 (53.9)	
Sedentary time spent (METs/day)			0.253
≤ 300	56 (54.4)	125 (60.7)	
> 300	47 (45.6)	81 (39.3)	
WHO recommendations on physical activity			0.053
Meet the recommendation	36 (35.0)	98 (47.6)	
Not meeting the recommendation	67 (65.0)	108 (52.4)	

^*^p-value for McNemar's Chi-squared test statistic

^**^The variable with more than two categories, *p*-value for McNemar-Bowker test statistic

^a^METs: metabolic equivalents

### Univariable conditional logistic analysis

Univariable conditional logistic regression analysis was conducted to quantify the magnitude of the unadjusted associations of various demographic, dietary and lifestyle factors and comorbidities with CRC status (Table 3).

**Table 3:** Univariable conditional logistic regression analysis of factors associated with colorectal cancer status in a hospital-based matched case-control study, Kuwait

Variable	Unadjusted matched OR^*^	95% CI^**^	*p*-value
Marital status (vs. single)			0.739
Married	1.41	0.50–4.01	
Divorced/widowed	1.65	0.45–6.03	
Education level (vs. Bachelor and higher)			0.025
< High-school	2.18	1.16–4.10	
High-school/diploma	1.03	0.58–1.82	
Family history with CRC (yes vs. no)	1.77	0.90–3.50	0.096
Red meat intake (days/ week) (vs. < 1 day/week)			< 0.001
1–3 days/week	4.04	2.10–7.84	
4–6 days/week	9.90	3.87–25.35	
Fruits and vegetables intake (days /week) (vs. Daily)			< 0.001
Often	2.65	1.54–4.56	
Rarely (never/< 1 a day)	16.35	4.52–59.16	
Egg(s) intake (days /week) (vs. < 1 day/week)			0.007
1–6 days/week	2.46	1.29–4.71	
Everyday	3.44	1.37–8.67	
Total PA per week (METs/ week) ^a^(≤ median (520) vs. > median (520)	1.81	1.12–2.94	0.014
Sedentary time spent/day (METs) (> 300 vs. ≤ 300)	1.32	0.8–2.172	0.272
WHO recommendation on PA (No vs. yes)	1.70	1.03–2.80	0.036
Maximum BMI ^b^ (vs. Normal)			< 0.001
Pre-obese	0.90	0.42–1.92	
Obese	2.50	1.20–5.14	
Diabetes history (yes vs. no)	0.31	0.17–0.57	< 0.001
History with hypercholesterolemia (yes vs. no)	0.36	0.21–0.63	< 0.001
Constipation (vs. Never)			< 0.001
Rarely	0.62	0.32–1.80	
Once in a month	1.81	0.85–3.86	
Every/week	3.19	1.55–6.56	

* *OR* odds ratio, ** *CI* confidence interval, ^a^*PA* physical activity, *METs* metabolic equivalents

^b^*BMI* body mass index

#### Family history of CRC

Univariable conditional logistic regression analysis showed that compared to controls, cases were more likely to report positive family history of CRC (mOR_unadj_ = 1.8; 95% CI: 0.9–3.5).

#### Dietary factors

The cases compared to controls tended to consume eggs 1–6 days a week (mOR_unadj_ = 2.5; 95% CI: 1.3–4.7) or every day (mOR_unadj_ = 3.4; 95% CI: 1.4–8.7). Similarly, cases compared to controls frequently consumed red meat 1–3 days/ week (mOR_unadj_ = 4.0; 95% CI: 2.1–7.8; *p* = 0.007) or 4–6 days/ week (mOR_unadj_ = 9.9; 95% CI: 3.9–25.4). Additionally, cases compared to controls tended to consume fruits/ vegetables less frequently (*rarely*, mOR_unadj_ = 16.4; 95% CI: 4.5–59.2; *often*, mOR_unadj_ = 2.7; 95% CI: 1.5–4.6).

#### Physical activity

Lack of required ‘total physical activity (METs)’ (mOR_unadj_ = 1.8; 95% CI: 1.1–2.9), unmet WHO recommended level of physical activity (mOR_unadj_ = 1.7; 95% CI: 1.03–2.8), or obesity (BMI ≥ 30) (mOR_unadj_ = 2.5; 95% CI: 1.2–5.1) were significantly more common among cases than controls.

#### Comorbidities

The cases compared to controls were more likely to report of having constipation nearly every week (mOR_unadj_ = 3.2; 95% CI: 1.6–6.6). However, two other morbidities considered were less common among cases than their matched controls including history of diabetes mellitus type II (mOR_unadj_ = 0.3; 95% CI: 0.2–0.6) or hypercholesterolemia (mOR_unadj_ = 0.4; 95% CI: 0.2–0.6).

### Multivariable conditional logistic regression model

Table 4 presents the risk factors significantly (*p* < 0.05) and independently associated with CRC status in multivariable conditional logistic regression model. After adjusting for effects of other variables in the model, cases were 4.3 times more likely to have had attainted obesity in their lifetime compared to controls (mOR_adj_ = 4.3; 95% CI: 1.6–11.4). Additionally, compared to controls, CRC cases rarely (or never) consumed fruits and vegetables (mOR_adj_ = 20.8; 95% CI: 4.4–99.5), or tended to consume red meat 2–3 days a week (mOR_adj_ = 3.8; 95% CI: 1.6–8.7) or more than 4 days a week (mOR_adj_ = 9.4; 95% CI: 2.5–35.4). Moreover, compared to controls, CRC cases frequently (nearly every week) suffered from constipation (mOR_adj_ = 5.6; 95% CI: 1.9–16.5). However, history of two other comorbidities considered were significantly less common among CRC cases than controls *including* hypercholesterolemia (mOR_adj_ = 0.3; 95% CI: 0.2–0.7) or diabetes mellitus type II (mOR_adj_ = 0.4; 95% CI: 0.2–0.8) indicating risk reduction by 70% and 60% respectively.

**Table 4:** Multivariable conditional logistic regression model of factors associated with colorectal cancer status in a hospital-based matched case-control study, Kuwait

Variable	Adjusted matched odds ratio	95% confidence interval	*p*-value
Maximum BMI^a^ ever attained			0.001
Normal	1.00	Ref	
Pre-obese	1.01	0.36–2.85	
Obese	4.30	1.62–11.37	
Fruits and vegetables intake			0.001
Daily	1.00	Ref	
Often	1.72	0.84–3.53	
Rarely (or never)	20.80	4.35–99.46	
Red meat intake (days/ week)			0.001
≤ 1	1.00	Ref	
2–3	3.76	1.63–8.67	
≥ 4	9.35	2.47–35.35	
Constipation history			0.005
Never	1.00	Ref	
Rarely	0.79	0.34–1.84	
Once a month	1.33	0.49–3.67	
Every/week	5.60	1.91–16.48	
Hypercholesterolemia history			0.005
No	1.00	Ref	
Yes	0.34	0.16–0.72	
Diabetes history			0.016
No	1.00	Ref	
Yes	0.35	0.15–0.82	

^a^*BMI* body mass index

## Discussion

CRC is regarded as one of the markers of the cancer transition, replacing infection-related cancers in countries undergoing rapid societal and economic changes together with other cancers predominantly linked to western lifestyles that are being adapted in high-income countries [19–21]. This hospital-based matched case-control study examined the association between CRC and dietary factors, maximum BMI ever attained, physical activity, comorbidities (constipation, diabetes, hypercholesterolemia), NSAIDs (non-steroidal anti-inflammatory drugs) use, tobacco smoking (cigarettes, hookah), alcohol drinking and family history of CRC in Kuwait. The multivariable conditional logistic model showed that being obese, frequent (2 or more days per week) consumption of red meat, rare (or never) consumption of fruits and vegetables, frequently (almost every week) having constipation were statistically significantly and independently associated with CRC risk. However, diabetes and hypercholesterolemia had significant inverse relationship with CRC risk in this study.

In this study, CRC cases were more than four times as likely to have been obese in their lifetime as were controls. A meta-analysis of 30 prospective studies showed that with every 5-unit increase in BMI, there was statistically significant increase in the CRC risk which was more pronounced in men (30%) than women (12%). The exact biologic mechanism(s) underlying the association between obesity and increased CRC risk is unclear, but seems to involve changes in the metabolism of endogenous hormones, including insulin, insulin-like growth factors, sex steroids, and possibly adipocyte derived factors such as leptin and adiponectin [22]. Obesity has been shown to be related to insulin resistance, to hyperinsulinemia, and to the development of diabetes type II [23]. Furthermore, it has been shown that high circulating concentrations of insulin and C-peptide (a marker of pancreatic insulin secretion) [24–26], and diabetes [27] were associated with increased CRC risk.

This study found that frequent consumption of the red meat during a week was significantly associated with increased odds of CRC in this population. Though a case-control study (281 cases and 566 controls) showed no relationship between red meat, total meat and other types of meat consumption and CRC risk [28], the finding of this study is consistent with the results on the significant association of red meat consumption and CRC risk reported from few countries in the Eastern-Mediterranean region including Saudi Arabia [29], Jordan [30], and Egypt [31]. Furthermore, a pooled relative risk from a dose-response meta-analysis of sixteen epidemiologic studies (9 case-control and 13 cohort studies) showed 36% more CRC risk with daily consumption of 170 g of red meat [32–34]. Another relatively recent dose-response meta-analysis of 13 prospective studies showed 22% increased CRC risk associated with red meat consumption (highest vs. lowest intake) [32]. There are several concurrent and/or alternate biologically plausible underlying mechanisms through which excessive red/processed meat consumption influences the CRC risk including; i) the formation of mutagenic heterocyclic amines and polycyclic aromatic hydrocarbons during the cooking process of the meat at high temperature and both are considered as carcinogenic compounds [35, 36]; ii) Nitrite is another carcinogenic compound available in some processed meat and transformed to *N*-nitroso in the colon [35–37]; iii) the bioavailability of heme iron in meat, which increases the development of *N*-nitroso, and iv) the availability of cytotoxic alkenals which are formed from fat peroxidation in the intestinal epithelium thus increasing intestinal inflammation [35, 36, 38], which may generate extensive and wide cascade of high level of malignant development [37]. Despite few conflicting results, accumulating epidemiological evidence seems to satisfy a few core Bradford Hill’s criteria for causal relationship between frequent red meat consumption and CRC risk including temporality, consistency, strength of association and biological plausibility. Therefore, public awareness campaign is likely to substantially reduce the CRC risk in this and other settings in the region.

In this study, we found a significant association between infrequent / low consumption of fruits, vegetables and CRC risk, which is consistent with findings of three other studies from the neighboring countries in the region [29–31]. Additionally, in many European countries, obesity, low intake / or less frequent consumption of fruit and vegetables have been consistently shown as significant risk factors for an increased CRC risk [39]. The biological mechanisms by which frequent consumption of fruits and vegetables accord the protection against cancers depend on phytochemicals in fruits and vegetables which modulate progression of different cancers, such as flavonoids which induce apoptosis. Furthermore, phytochemicals control the metabolism of carcinogens and inflammation, for example, isothiocyanates, flavonoids, resveratrol, and proanthocyanidins have shown anti-inflammatory activity [40]. Additionally, antioxidants in fruits and vegetables tend to decrease the cellular damage caused by reactive oxygen species which may cause cancer [40]. Finally, availability of fiber in fruits and vegetables help to increase bulk of stool, and reducing transit time over the gut, thus attenuating carcinogens [40].

History of frequent (i.e. once a week compared to never) constipation was significantly associated with CRC risk in this study. This result concurs with those of other case-control studies which had shown positive association between chronic constipation and CRC risk [11, 41–44]. The association between constipation and CRC risk is linked to longer transits times in the colon, resulting into an increased interaction between intestinal mucosa and concentrated carcinogenic agents, such as bile acids, fecapentaenes, and ammonium acetate in the lumen [41, 42]. Constipation is commonly prevalent in Kuwait more so in females than males [45]. Efforts to avoid the constipation may help minimize the CRC risk in this and other similar populations.

In this study, history of hypercholesterolemia diagnosis was less common among cases than controls (22.3% vs. 43.2%). This is consistent with an inverse association between serum cholesterol and CRC risk frequently observed in past studies [46–48]. It has been shown that statins use was significantly associated with a modest reduction in CRC risk. Since statin acts as a cholesterol lowering agent, thus reduces the level of low-density lipoprotein, and prevent coronary heart disease [47]. Also, it has a chemo-preventive ability in reducing tumor growth and angiogenesis, enhancing immunity, reducing metastatic potential, and increasing the likelihood of anti-cancer effects of some cytokines [46]. Additionally, in this study, history of diabetes mellitus type II diagnosis was less common among cases than controls (18.4% vs. 39.8%). Published literature revealed that metformin use by the patients with diabetes was associated with reduction in CRC risk [49–51]. Anti-cancer effect of metformin is still unresolved [51], however, it has been argued that metformin has anti-cancer activity through inhibiting cancer cell proliferation and metabolism [51]. Additionally, it has angiogenesis effect through inhibition of mammalian target of rapamycin and activation of adenosine monophosphate-activated protein kinase [49, 51]. In contrast, few other studies showed non-significant association between metformin use and reduced CRC risk in patients with DM type II [52, 53]. In this study, we did not actually record the use of statins and metformin among cases and controls, however, since these two morbid conditions i.e. past diagnosis of hypercholesterolemia and diabetes mellitus type II were more common among controls than cases, resultantly higher proportions potential users of statins and metformin among controls might have cancelled the effects of these two morbid conditions. Alternatively, the proportions of statins and metformin users both among cases and controls could be same, which might have annulled the effects of these two morbid conditions in two study arms. However, the resolution of these conflicting results needs further investigations.

In this study, alcohol consumption was not significantly associated with CRC status. Similar to the finding of this study, case-control studies from Egypt [31], Thailand [54, 55] and Canada [54, 55] could not find significant relationship between alcohol drinking and CRC risk. In contrast, some other case-control and cohort studies have shown statistically significant association between alcohol drinking and CRC risk both in men and women [56]. The alcohol for public consumption is not available in Kuwait’s consumers’ markets and admittance to alcohol drinking both by CRC cases and controls seems odds against the religious belief and therefore this exposure might have been somewhat underestimated that resulted in non-significant association with CRC risk in this study. Nevertheless, this opposing evidence ought to be addressed in further investigations.

This study did not show a meaningful relationship between smoking and CRC risk. Whereas, several recent case-control and cohort studies showed that smoking had a significant role in CRC development [57–59]. The non-significant association between tobacco smoking and CRC risk in our study could possibly be due to potential underreporting of smoking by the participants of this study. Face-to-face interview as a data collection procedure is known to suffer this sort of setback [60, 61]. Alternatively, it can be reasoned based on the results of a meta-analysis of cohort studies, wherein it has been argued that the effect of tobacco smoking on colorectal mucosa is smaller than the one on lung and esophageal mucosa and this relationship was consistent across the cohort studies regardless of the country and the region. Additionally, it was further debated that since age at initiation and duration of smoking widely varied across the cohort studies included in the meta-analysis, the effect of smoking on CRC risk possibly was attenuated [62]. Additional empirical evidence is needed to confirm or refute such a relationship between tobacco smoking and CRC risk.

The strengths of this study include, firstly, enrolled cases were somewhat a representative sample of CRC cases, but the controls were not randomly sampled from general population, instead selected from tertiary-care hospitals spread out across the State of Kuwait. Thus, controls can be regarded as typical of Kuwaiti population, perhaps rationalizing the generalizability of results beyond the study population. Secondly, all the CRC cases were diagnosed in a relatively narrow time window before their enrollment in the study, therefore, presumably were comparable with the incident CRC cases. Thus, this feature of CRC cases’ selection might have helped in minimizing the recall bias in exposures’ assessment. Few limitations of this study should be considered in interpretation of the results; *First*, being a retrospective study, recall bias could be an issue in exposures' assessment more so in controls than cases. Since cases tend to remember history of exposures better than controls. However, we tried to minimize this bias in exposures' assessment during a time window prior to the date of CRC diagnosis or pseudo-diagnosis for cases and controls respectively. *Second*, we collected the data both from cases and controls through face-to-face interview, thus there was a possibility of interviewee bias, since the respondents might have withheld answers to some of questions pertaining to lifestyle including smoking, alcohol consumption and or physical activity etc. However, the interviewer was trained in interviewing technique and was cautious about this aspect of exposures' assessment, hence tried hard to pose the questions to cases and controls in a comparable manner to ensure the accuracy of sought information. *Third*, since this was hospital-based case-control study, thus the role of Berkson’s bias cannot be ruled out. This bias tends to make risk factors’ distributions similar in case and control groups, thus attenuating the strength of associations of interest between the outcome (case-control status) and studied risk factors [63]. Nonetheless, most adjusted associations estimated as multivariable mORs relating potential risk factors and CRC status were substantially large as oppose to being absent or weak in magnitudes in this study. Therefore, even if the Berkson’s bias has crept in the data due to hospitals-based controls, its role seems to be little in this evaluation. *Fourth*, we found that cases compared to controls had less frequent history of physician-diagnosed diabetes mellitus type II (18.4% vs. 39.8%) and physician-diagnosed history of hypercholesteremia (22.3% vs, 43.2%). Presumably therefore, metformin and statins use was more common among controls than cases resulting in inverse associations of these morbid conditions with CRC risk. Though we outlined above some plausible mechanistic pathways for the observed inverse associations between these morbid conditions and CRC risk, yet we did not have the empirical data on the use of these two drugs to support this contention. Future studies on this question should consider this aspect at planning stage. *Fifth*, CRC cases were histopathologically confirmed, whereas, the controls in this study did not undergo same diagnostic/ screening procedures to rule out the CRC positive status, rather their CRC status was self-reported. Such differential diagnostic/ screening procedures for the assessment of disease status in cases and controls might have introduced information bias in the data. However, we selected the controls from among the individuals who were visiting the general hospitals for seeking care for medical conditions other than that of gastrointestinal tract. Therefore, any information bias if crept in the data must be very minimal. Nevertheless, any future study on this question should consider enrollment of controls from among the individuals who undergo routine CRC screening and turnout to be CRC negative. Final, some of the cases soon after CRC diagnosis travelled abroad for treatment, we might have missed out such cases from enrollment in this study. However, the proportion of such CRC patients presumably was not large enough to impair the parameters’ estimates in this study.

In summary, obesity, excessive red meat consumption and infrequent fruits/vegetables intake were associated with an increased CRC risk**.** Overcoming identified pitfalls in dietary pattern and maintenance of healthy weight may help minimize CRC risk in Kuwait and perhaps other countries in the region. Further studies on the genetic basis in conjunction with the life styles and dietary factors may unravel their joint contributions to CRC risk and furnish tools for curtailing CRC risk in this and other similar populations.

## Abbreviations

CI: Confidence interval
CRC: Colorectal cancer
KCCC: Kuwait Cancer Control Centre
METs: Metabolic equivalents
mOR: matched odds ratio
mOR_adj_: Adjusted matched odds ratio
mOR_unadj_: unadjusted matched odds ratio
NSAIDS: Non-steroidal anti-inflammatory drugs
OR: Odds ratio
